# Arsenic Exposure within the Korean Community (United States) Based on Dietary Behavior and Arsenic Levels in Hair, Urine, Air, and Water

**DOI:** 10.1289/ehp.11827

**Published:** 2008-12-08

**Authors:** Bill Cleland, Ami Tsuchiya, David A. Kalman, Russell Dills, Thomas M. Burbacher, Jim W. White, Elaine M. Faustman, Koenraad Mariën

**Affiliations:** 1 Office of Environmental Health Assessments, Washington State Department of Health, Olympia, Washington, USA;; 2 Department of Environmental and Occupational Health Services and; 3 Institute for Risk Analysis and Risk Communication, University of Washington, Seattle, Washington, USA

**Keywords:** air, arsenic, exposure, hair, inorganic, intake, shellfish, urine, water

## Abstract

**Background:**

Determining arsenic exposure in groups based on geographic location, dietary behaviors, or lifestyles is important, as even moderate exposures may lead to health concerns.

**Objectives/Methods:**

The Korean community in Washington State, represents a group warranting investigation, as they consume foods (e.g., shellfish, rice, finfish, and seaweed) known to contain arsenic. As part of the Arsenic Mercury Intake Biometric Study, we examined the arsenic levels in hair and urine along with the diets of 108 women of childbearing age from within this community. Arsenic levels in indoor air and drinking water were also investigated, and shellfish commonly consumed were collected and analyzed for total and speciated arsenic.

**Results:**

The six shellfish species analyzed (*n* = 667) contain total arsenic (range, 1–5 μg/g) but are a small source of inorganic arsenic (range, 0.01–0.12 μg/g). Six percent of the individuals may have elevated urinary inorganic arsenic levels (> 10 μg/L) due to diet. Seaweed, rice, shellfish, and finfish are principal sources for total arsenic intake/excretion based on mass balance estimates. Rice consumption (163 g/person/day) may be a significant source of inorganic arsenic. Air and water are not significant sources of exposure. Hair is a poor biometric for examining arsenic levels at low to moderate exposures.

**Conclusions:**

We conclude that a portion of this community may have dietary inorganic arsenic exposure resulting in urine levels exceeding 10 μg/L. Although their exposure is below that associated with populations exposed to high levels of arsenic from drinking water (> 100 μg/L), their exposure may be among the highest in the United States.

The harmful effects of long-term exposure to arsenic in humans are well studied and widely recognized. There is worldwide concern about As, as everyone has some exposure through water, food, soil, and air, and because many people have sufficient exposure to cause measurable population increases in a variety of health problems. When investigating As exposure from drinking water or food, the inorganic forms of arsenic [arsenate (As^+5^) and arsenite (As^+3^)], which are considered to be among the most toxic forms, require our focus [Agency for Toxic Substances and Disease Registry (ATSDR) 2007; National Research Council (NRC) 1999]. Although some food intake data that address As exposure within populations are available, biometric data are most often used to assess exposure (Chew 1996; Hwang et al. 1997; Kalman et al. 1990; MacIntosh et al. 1997; Mohri et al. 1990).

Urinary As levels are considered the best indicator of exposure that occurred during the few days prior to sample collection (NRC 1999). Urinary As species combined [As^+5^, As^+3^, monomethylarsonic acid (MMA) and dimethylarsenic acid (DMA)] can reflect exposure to inorganic As, and average background rates from individuals in European countries and in the United States have been ≤ 10 μg/L (Andrén et al. 1988; Binder et al. 1987; Buchet et al. 1996; Foà et al. 1984; Jensen et al. 1991; Kavanagh et al. 1998; Kristiansen et al. 1997; Morse et al. 1979; NRC 1999; Smith et al. 1977; Trepka et al. 1996; Vahter and Lind 1986; Valkonen et al. 1983). Results for the U.S. population from the National Health and Nutrition Examination Survey (NHANES) suggest that the 50th percentile of urinary As species combined is approximately 6 μg/L (Caldwell et al. 2008). Exposures to elevated levels of As in drinking water in Taiwan and Argentina have resulted in 5- to 50-fold higher concentrations of these compounds in urine (Chiou et al. 1997; Vahter et al. 1995).

Hair As levels can be a useful indicator of long-term or chronic exposure when external contamination to hair is properly controlled (Harkins and Susten 2003). Persons with no known exposure to As have hair As levels ranging from 0.02 to 0.2 μg/g, whereas individuals exposed to elevated levels of As in drinking water have hair As levels ranging from 3 to 10 μg/g (Das et al. 1995; Kapaj et al. 2006; Koons and Peters 1994; Kurttio et al. 1998; Narang et al. 1987; Olguín et al. 1983; Paulsen et al. 1996; Raie 1996; Takagi et al. 1988; Valentine et al. 1979; Vienna et al. 1995; Wang et al. 1994; Wolfsperger et al. 1994).

To characterize populations with higher relative dietary As, groups consuming relatively large amounts of As-containing foods such as shellfish, seaweed, finfish, rice, and chicken require assessment. The Korean community from within Washington State, represents such a population and was examined to determine sources and extent of exposure to total As and inorganic As. This is the first analysis we are aware of that addresses As exposure within a population by linking biometric data with environmental data that include air, water, and food exposure, with the latter measured using both food frequency questionnaire (FFQ) and fish consumption survey (FCS) results.

## Methods

### AMIB Study and study population

Goals of the Arsenic Mercury Intake Biometric Study (AMIBS) included determining the extent of mercury exposure within the Japanese and Korean communities and the extent of exposure to As by the Korean community. Mercury exposure in these two populations is of importance, as both consumed fish in quantity and at levels higher than the national average. Arsenic exposure was considered of concern within the Korean population, as they consume shellfish to a greater extent than the Japanese while also consuming As-containing foods such as rice, finfish, and seaweed.

A detailed description of AMIBS has been published previously (Tsuchiya et al. 2008a,b). Informed consent was obtained from all study participants. Study design and materials were approved by the State of Washington Department of Social and Health Services Human Research Review Board.

### Arsenic-specific study outcome measures for the Korean participants

This article reports dietary data from the FCS and the FFQ, environmental As concentrations in indoor air and drinking water, and biomonitoring data (hair and urine As concentrations). In addition, tissue concentrations of total and speciated As were determined for the various types of shellfish consumed. Details on arsenicals used for analyses are available in Supplemental Material, Section 1, at http://www.ehponline.org/members/2009/11827/suppl.pdf.

The FCS was based on surveys previously used with Native American tribes, recreational fishers, Asian Pacific Islanders, and the general public ( Landolt et al. 1985; Mariën and Patrick 2001; Toy et al. 1996). Individual shellfish intake estimates were determined from each participant’s consumption behavior using data from the FCS. As part of the interview, participants were asked about their fish (finfish and shellfish) consumption. Demographic information was collected including smoking status, age, and weight. Participants were shown pictures with names, in English and Korean, of fish that are commonly consumed by Koreans as well as fish species commonly found in the Pacific Northwest. For each species consumed, the participant was asked to answer questions about frequency of consumption and serving sizes. Participants were asked if they consumed any fish species not listed.

The FFQ was a validated tool developed at the Fred Hutchinson Cancer Research Center (Kolar et al. 2005; Kristal et al. 1994; Patterson et al. 1999) and was augmented slightly for use in this study (e.g., addition of a section specific to food items reportedly consumed by the Korean population). FFQ data provided consumption rates for food items known to contain As such as rice and chicken.

Hair samples were collected from the nape of the neck and one additional location on the side of the head. The complete hair sample (nominally 100 mg) was then analyzed for total As using inductively coupled mass spectroscopy (ICP-MS) after hot-block digestion in HNO_3_-H_2_O_2_ [U.S. Environmental Protection Agency (EPA) 1992]. The average sample weight was 134 mg [standard deviation (SD) ± 30 mg]. The limit of quantification (LOQ) was 0.5 μg/g of hair.

Urine samples were collected in sterile polypropylene containers, and placed on ice during transfer to the lab. Total As was measured by ICP-MS after a 1:5 dilution of 1 mL urine and acidification to 5% HNO_3_. The LOQ was 2.5 μg/L urine.

Inorganic arsenic (sum of As^+5^ and As^+3^), MMA, and DMA in urine were determined by batch hydride generation with cryogenic trapping with atomic fluorescence detection (Kalman 1988). Arsenobetaine (AsB), arsenocholine, and arsenosugars are not detected by this method, and their total concentration is assumed to be the difference between the speciated forms and total As determined by ICP-MS. The LOQ for any species was 5 μg As/L urine.

Indoor airborne As concentrations were determined by analyzing charcoal and HEPA filter elements from air purifiers (Honeywell, QuietCare Permanent True HEPA Air Purifier, Model # 17000; Southborough, MA) that were provided to participants on a voluntary basis. Further detail are available in Supplemental Material, Section 2, at http://www.ehponline.org/members/2009/11827/suppl.pdf.

Data obtained from the Washington State Department of Health were used to identify the public water system providing drinking water to a participant’s home and to determine the average source water As values from 1990 to 2006.

### Shellfish (mollusk) sampling and analysis

People from many cultures and populations, including the Koreans, dig for and consume shellfish from Puget Sound (Washington State). Samples of six bivalve mollusk species (littleneck, butter, varnish, horse, and Eastern softshell clams, along with oysters) of legal size (*n* = 667) collected from 16 Puget Sound locations were analyzed for total As, DMA, MMA, and inorganic As. Some specimens were analyzed individually to determine variance within the population, whereas composite analyses were conducted to determine As concentrations from a larger sample at reduced cost. For Eastern softshell and horse clams, the siphon/mantle and viscera were analyzed separately. A total of 137 samples was analyzed, with individual analyses conducted for all species except varnish clam; composite values were determined for all species with the exception of Eastern softshell clam. Table 1 provides a synopsis by species and sample number. Specimens were collected, rinsed, and placed on ice at the beach and frozen at the laboratory. Samples were shucked, separated by siphon (with skin) and viscera if necessary, and rinsed prior to homogenization. Total As in tissues was quantified using ICP-MS after hot-block digestion in HNO_3_-H_2_O_2_ (U.S. EPA 1992). The quantification limit for total As was 0.02 μg/g tissue (wet weight).

Individual As species in shellfish were determined by HPLC–ICP-MS. Extraction was by the method of Hsiung and Huang (2006) and by proprietary HPLC and ICP-MS conditions developed by the manufacturer for a specialty As speciation column (G3288-80000 and G3154-65002 guard column) (Sakai and Wilber 2006). For the As species investigated, the quantification limit for As^+3^, MMA, and As^+5^ was 0.02 μg As/g wet tissue and for DMA and AsB was 0.05 μg As/g wet tissue.

Because As values in tissues in some studies are provided on a dry-weight basis, six samples of two shellfish species (butter and littleneck clams) were analyzed to determine wet-weight to dry-weight ratios. Aliquots of homogenate (5 g) were dried in a vacuum oven for 4 days at 80°C and then cooled in a desiccator prior to weighing.

### Inorganic arsenic intake from shellfish consumption

Exposure to inorganic As from shellfish was estimated for each individual by multiplying the individual shellfish consumption levels by the mean inorganic As concentration of each species. The inorganic As intake levels by species were then combined for all species to obtain a total daily individual inorganic As intake estimate.

The inorganic As levels used for little-neck clams, butter clams, and oysters were the average of the individual values obtained (0.08, 0.06, and 0.02 μg/g, respectively). Razor clams, mussels, and geoducks were not collected for this study because of limitations in digging season or physical accessibility. The average butter clam inorganic As level (0.06 μg/g) from this study was used for razor clam, whereas the littleneck clam value (0.08 μg/g) was used for mussels, as these are similar species of approximately equal size. For the geoducks, horse clam results from this study (0.12 μg/g) were used in one set of analyses, whereas results from Ostrom and coworkers (2007) (in which no inorganic As was detected in samples analyzed) were used in separate analyses for comparative purposes. For Eastern softshell and horse clams, siphon As values were used instead of siphon and viscera combined. Decapod crustaceans (i.e., shrimp, lobster, and crab) were not used in these calculations, as the predominant form of arsenic found in these species is AsB, not inorganic As (NRC 1999). Accordingly, we obtained no data for these species.

### Inorganic arsenic intake from rice and chicken consumption

The validated FFQ uses a series of questions to determine individual consumption of a broad range of food items, including rice and chicken, which are known sources of dietary As. The rice and chicken intake levels were multiplied by reported inorganic As contaminant levels to estimate individual inorganic As intake levels from these food items. Inorganic As levels used for rice and chicken were 0.1 μg/g and 0.08 μg/g, respectively. Both were derived from the literature, with detail provided in Supplemental Material, Section 3 (available online at http://www.ehponline.org/members/2009/11827/suppl.pdf).

### Total arsenic intake

Data from the FCS and FFQ were combined to estimate mean total daily As intake. These results are based on 89 of 108 Koreans for whom all data were available (one individual did not complete the FFQ) and who reported consuming > 600 Kcal/day (*n* = 18 reported consuming < 600 Kcal/day). Total energy intake was used as the criterion to determine if the FFQ was completed adequately, and in this study, this resulted in 18 individuals being omitted as outliers (Willett 1998). Ingestion rates of rice and seaweed were determined from the FFQ and crab, shrimp, finfish, and shellfish (mollusks) were determined from the FCS. Ingestion rates for each of the food items were multiplied by total As contaminant levels to derive individual total As intake levels. The total As level for each food type was determined from the literature, with the exception of the shellfish investigated in this study. Values with ranges are documented in Table 2. Details of how these values were obtained are available in Supplemental Material, Section 4, at http://www.ehponline.org/members/2009/11827/suppl.pdf.

For this population, 75% of the finfish intake came from eight species (Tsuchiya et al. 2008b). Species-specific total As exposure from finfish was estimated by multiplying the median total As concentration for each of these species by the intake rate of each individual. Combined finfish exposure for each individual was determined by summing the species-specific exposures. Because the eight species represented only 75% of finfish intake, the calculated exposure from these eight species was multiplied by 1.33 to estimate the full exposure (100%) of total As from finfish consumption. This was considered an acceptable approach, as more than 50 species of fish were consumed by the community. Details regarding the median total As values used for the eight species are provided in the Supplemental Material, Section 5 (available online at http://www.ehponline.org/members/2009/11827/suppl.pdf).

Total As intake levels from shellfish (mollusks) were determined in a manner similar to that used to determine inorganic As (described above); ingestion rates for the shellfish species collected along with three other mollusks consumed (geoduck, mussels, and razor clam) were used with total As levels from tissue sample data to estimate individual total As intake on a daily basis. Average values obtained from the individual samples for littleneck clam (3.7 μg/g), butter clam (4.9 μg/g), and oyster (1.3 μ/g) were used to derive total As intake levels. Accordingly, the razor clam, mussel, and geoduck values used were 4.9, 3.7, and 1.4 μg/g, respectively.

### Statistical analyses

Analysis of variance (ANOVA) with post-hoc comparisons using the Tukey HSD test were performed to compare the arsenic intake from shellfish and rice consumption across age groups. For all age-based analyses, individuals were combined to form four groups (< 30, 30–34, 35–39, and 40 years of age and older) because of the small number of subjects in some age categories. We used Student’s *t*-test to compare means of inorganic As levels, as well as total As levels, within species based on sample type (individual or composite). Regression analyses were performed to compare rice, chicken, seaweed, and shellfish intakes with urine values for As on an individual basis. Statistical analyses were performed using a significance level of 5% (*p* < 0.05) and completed using Stata (Stata Corporation, College Station, Texas), Excel (Microsoft Corporation, Redmond, WA) and SPSS (SPSS Inc., Chicago, IL).

## Results

The total As and/or As species content of food stuffs, urine, air, water, and hair was obtained from the literature or originated with this study. Air, hair, and urine values were measured as part of this study. For drinking water, finfish, crab, rice, chicken, seaweed, shellfish (mollusks), and shrimp values were obtained from the literature.

### Air As concentrations

Of the 108 Korean participants in the study, 32 took home air filter units; units were kept an average number of 102 days (SD ± 33). The average concentration of As in air for participants was 0.4 ng/m^3^ (SD ± 0.3; 95% confidence interval, 0.3–0.5). A control unit placed in a very active area (bedroom/play area of a 12-year-old) and left running for 42 days had an air As concentration of 2.7 ng/m^3^.

### Water As concentrations

The maximum contaminant level (MCL) for arsenic in drinking water is 10 μg/L (U.S. EPA 2006). Data for 2 of the 108 individuals were not obtained, as they received water from individual wells. For the remaining participants, the average water As concentration was < 2 μg/L, and none had source water exceeding the MCL.

### Shellfish total As and inorganic As levels

Table 1 provides total and inorganic As levels on a wet-weight basis for the six bivalve mollusks we sampled. The ratio of inorganic to total As was less than 1:10 for all species. Only the horse clam siphons had inorganic As levels that approached 10% of the total As levels, as 8.5% of the total As value of 1.4 μg/g (SD ± 0.8) was inorganic As (0.12 μg/g ± 0.06). Viscera from several individual horse clams had no detectable inorganic arsenic levels, resulting in an average viscera value half of that observed for the siphon samples.

For total As and inorganic As, the mean individual and composite levels were significantly different for the littleneck and butter clams but not for the oysters. For the little-neck and butter clams, the individual mean As values were greater than the composite mean values. These outcomes were not unexpected, as the oysters used for individual analysis were taken from the total sample collected, with the remaining being used for composite sample analysis. With the littleneck and butter clams, only large individuals were used for individual analysis, whereas a range of clam sizes was used for composite analysis. The difference in mean values observed between the composites and individuals for butter and littleneck clams may be a size- or age-dependent effect. In this study, we used mean individual As values for determining individual exposure.

To allow for the total As and inorganic As wet-weight values obtained in this study to be compared with dry weight values determined from other studies, we examined the percent dry weights across six samples of two shellfish species (butter and littleneck). Percent dry weights were 18.4 ± 1.0 and 18.3 ± 2.8 (mean ± SD) for butter and littleneck clams, respectively, resulting in a wet weight to dry weight ratio of approximately 5:1 for the combined samples.

### Inorganic As intake from shellfish (mollusk) consumption

Based on the consumption survey data, the average inorganic As intake from the shellfish (mollusks) consumed in this population was 0.7 μg/day (SD ± 1.0). This assumed that the inorganic As level in geo-duck was equal to the horse clam siphon level (0.12 μg/g) observed in this study. However, the average intake level fell to 0.5 μg/day (SD ± 0.8) when using results from Ostrom and coworkers (2007), who found no detectable inorganic As in their geoduck samples. The range of inorganic As intake for individuals remained the same and was 0–5.9 μg/day. Age-based analysis using ANOVA with the Tukey HSD test indicated no significant difference in total shellfish consumption across age groups (Tsuchiya et al. 2008a). Regression models were established using individual as well as log-transformed individual urine inorganic As values and estimated individual shell-fish inorganic As intake levels. Models were also made using the sum of As species (inorganic As along with DMA and MMA) levels observed in urine and compared with estimated shellfish inorganic As intake. Models were also established using creatinine-normalized values. Although significant results were obtained, none of the models explained more than 1–2% of observed variability in urine inorganic As or DMA levels.

### Inorganic As intake from rice and chicken consumption

Mean intake of chicken and rice was 21 (SD ± 27) and 163 (SD ± 157) g/person/day, respectively. With a rice inorganic As level of 0.1 μg/g, the mean inorganic As intake was 16.3 ± 15.7 μg/person/day. For chicken, the mean inorganic As intake level was 1.7 μg/day (SD ± 2.2). ANOVA with post hoc comparisons using the Tukey HSD test showed no significant differences in chicken consumption across age groups, but did show a difference in rice consumption between those < 30 and > 40 years of age. Those < 30 years of age consumed 115 g/day of rice (*n* = 37), whereas those > 40 years of age consumed more than twice that amount (260 g/day; *n* = 19). Regression analyses investigating the relationship between chicken consumption and As species found in urine were conducted, but no correlations explaining the observed variability in urine inorganic As were found. Regression models comparing the urinary sum of As species (inorganic As, DMA, and MMA) for each individual (as well as log-transformed sums) and individual inorganic As intakes from rice consumption yielded statistically significant outcomes. However, as for chicken and shellfish, only a small portion of the observed variability in urine As levels (2–3%) was explained by the models.

### Hair As concentrations

Of the 108 participants, all but 10 individuals had hair As values at or below the detection limit of 0.5 μg/g (data not shown). Of the remaining 10, all had hair As values < 1.0 μg/g. Because fewer than 10% of the samples were properly quantified, statistical analyses were not conducted, as all sample statistics would be biased.

### Urine As concentrations

Levels of total As and As species were determined for the 67 individuals who provided urine samples of the total of 108 participants. Individual urine creatinine levels were also measured to make available adjusted urine As levels and as a check (< 50 mg/dL or > 300 mg/dL creatinine) for tampering by dilution with water and the extremes of diuresis, in which case the excretion measure may not be a reliable indicator of exposure [American Conference of Industrial Hygienists (ACGIH) 2001]. Table 3 provides unadjusted and creatinine-normalized urine data, with the sum of As species column depicting the combination of DMA, MMA, As^+3^, and As^+5^. The mean sum of As species was 30.6 μg/L urine and 37.2 μg/g creatinine. Mean total As values were 127.3 μg/L urine and 152.1 μg/g creatinine. Geometric mean (GM) values were also determined, because the urine As distributions showed positive skew (Table 3). Average MMA levels were low (1.2 μg/L urine and 1.5 μg/g creatinine), whereas the average DMA levels were approximately one order of magnitude greater than the inorganic As levels (27 μg/L urine and 33 μg/g creatinine for DMA versus 2.4 μg/L urine and 2.9 μg/g creatinine for inorganic As). Mean unadjusted levels for total As and sum of As species were lower prior to creatinine normalization. A major factor for this difference was that there were 19 individuals with creatinine levels at or below 50 mg/dL. For all As species analyzed, SDs were large relative to the mean, indicating that the As species as well as total As distributions were broad within this group.

To identify individuals possibly having elevated intake of inorganic As due to diet, we evaluated DMA, inorganic As, and sum of As species in urine. MMA levels were not included in this analysis, as few individuals had urinary MMA levels above the detection limit. Individuals with creatinine levels between 50 and 300 mg/dL and with sum of As species > 25 μg/L were examined first, as they represented those with the highest exposures. Eleven individuals that met these criteria, but for each of these individuals, the DMA level represented > 90% of the sum of As levels. Accordingly, the DMA was not all from metabolized inorganic As, but also from food sources containing DMA. Further analysis indicated that of the 67 participants, four (6%) had urinary inorganic As levels greater than the sum of As species levels observed in Europe and the United States (10 μg/L) while also having sum of As species elevated compared with the DMA level (e.g., a minimum 30% difference between the two values). For these 6%, dietary sources could be providing heightened inorganic As exposure.

### Mass balance: total As intake versus excretion

The mean sum of total As excreted in urine, assuming an excretion rate of 1.5 L/day, was 191 μg/day. Mass balance was estimated in an endeavor to determine which food sources provided the majority of the total As intake. Estimated mean daily total As intake levels were derived for several food types based on consumption rates and median total As level in the food item (Table 2). Estimated mean total As intakes for crab and shrimp were 21 μg/day and 7 μg/day, respectively. The estimated intake was 39 μg/day of total As from shellfish (mollusks) consumed. The sum of these three shellfish categories yielded a total As intake of 67 μg/day. Additional sources of total arsenic from other shellfish species in this population is small, as the estimated mean consumption rate for the crab, shrimp, and mollusks (24.4 g/day) is nearly equivalent to the estimated mean consumption rate for all shellfish species (24.8 g/day). The estimated median total As intakes from rice, seaweed, and fin-fish are 39 μg/day, 10 μg/day, and 79 μg/day, respectively. The sum of crab, shrimp, shellfish (mollusks), rice, seaweed, and finfish intake levels yields a total As intake of 195 μg/day and is similar to the mean value of total As excreted (Table 2).

#### Sum of As species consumed from shellfish (mollusks) versus excreted in urine

Arsenic species data from the shellfish collected for this study (Table 1) and for three other clam species consumed (geoduck, mussels, and razor clam) were used to derive an estimated intake of As^+3^, As^+5^, DMA, and MMA (Table 4). This value was tabulated for the total sample size (*n* = 108) as well as for those individuals from whom urine samples were obtained (*n* = 67). Estimated intakes of these As species were 2.0 μg/day (SD ± 2.7) and 1.7 μg/day (SD ± 2.5) for the total cohort and for those who provided a urine sample, respectively. Mean sum of As species excreted in urine, based on an excretion amount of 1.5 L/day, was also determined and resulted in a value of 47.9 μg/day (SD ± 40.9). Sum of species values were based on intake of DMA, MMA, As^+3^_,_ and As^+5^. The intake estimates for these As species from the consumption of shellfish indicates that this food type represents only a small fraction (≈ 4%) of the sum of species excreted.

## Discussion

Long-term moderate exposure to As within a population may lead to adverse health outcomes. Because exposure to As is dependent on a specific population’s lifestyle, location, and/or dietary behavior, we examined a group considered to be moderately exposed, as they consume various foods known to contain As. In conducting this work, we used a multi-faceted approach to determine the extent of exposure to As within the Korean population. Further, the use of hair samples as a viable tool for determining As levels in less extreme exposures was evaluated, and we endeavored to determine the sources of As exposure in the community.

Results indicate that air and water are not significant sources of As exposure. Presently, there is no reference concentration for exposure to inhaled arsenic. The U.S. Environmental Protection Agency (U.S. EPA) has established a quantitative estimate of carcinogenic risk from inhalation that is available in its Integrated Risk Information System (U.S. EPA 2007). The model uses a linear extrapolation method to calculate risk estimates at various concentrations. For example, the U.S. EPA risk estimate for lifetime exposure to 2 ng As/m^3^ is one added lung cancer death for every 100,000 individuals exposed. The air As concentrations were intended to aid in controlling for airborne deposition of As onto hair and to determine what percentage of the individual body burden came from inhalation. However, none of the participants had measurable in-home indoor air As levels in excess of 2 ng As/m^3^, with only one location slightly exceeding this air As concentration (control; bedroom of a 12-year-old). Drinking water As values were analyzed to determine if As was a significant source of exposure and to more accurately determine hair As levels by controlling for arsenic deposition on hair from washing and bathing. Consuming drinking water at a rate of 1.5 L/day will not appreciably increase the As level in urine in this population. The percentage of inorganic As contributed by this source is small and similar to that obtained from clam consumption.

Although the analysis of shellfish (mollusks) indicate that total As is present, only a small fraction is inorganic As. Clam species inorganic As concentrations were all less than 10% of the total As levels, with the highest being 8.5% and with many species having only 2–3% inorganic As. This suggests that these shellfish are not a large source of inorganic As in this population. This outcome does not change in spite of recent findings by Ostrom and coworkers (2007). They observed total As levels in geoduck to be greater than those observed in horse clams for this study, but found no detectable inorganic As levels in the geoduck samples analyzed. The supposition that shellfish is not a large source of inorganic As is further supported by intake estimates. Although this population consumes (≈ 25 g/day) more than the general population (10–18 g/day), the average inorganic As intake from the shellfish was low (0.5–0.7 μg/day) [Office of Environmental Health Hazard Assessment (OEHHA) 2001].

Compared with the various studies investigating background levels of the sum of As species in urine, the Korean participants who provided urine samples for this study had an average value (≈30 μg/L) higher than that observed in European countries and in the United States (≤ 10 μg/L) but below that observed in Japan (≈50 μg/L) (Buchet et al. 1996; Foà et al. 1984; Gottlieb et al. 1993; Kalman et al. 1990; Kavanagh et al. 1998; Kristiansen et al. 1997; Pollisar et al. 1990; Trepka et al. 1996; Vahter et al. 1995; Yamauchi et al. 1989). U.S. population data from NHANES indicate that the 50th percentile for sum of species in urine was 6.0 μg/L (although the estimate may be slightly biased), and the 95th percentile was 18.9 μg/L (Caldwell et al. 2008). The 50th percentile value determined from our study is greater than 3-fold the 50th percentile seen nationally and exceeds the 95th percentile level. The 95th percentile sum of species value is significant within the NHANES distribution, as the remaining 5% represents many individuals. Although the sample size of 67 individuals who provided urine samples is too small to represent the whole of the Korean community, our results suggest that this population may be contained within the remaining 5th percentile of the NHANES distribution representing the U.S. population. Also, based on urinary As (inorganic and sum of species) results, this community may have a percentage (6%) of individuals with elevated inorganic As exposure due to dietary behavior.

Individuals exposed to ambient levels of As have hair As levels ranging from 0.02 to 0.2 μg/g ( Kapaj et al. 2006; Koons and Peters 1994; Kurttio et al. 1998; Narang et al. 1987; Olguín et al. 1983; Paulsen et al. 1996; Raie 1996; Takagi et al. 1988; Valentine et al. 1979; Vienna et al. 1995; Wang et al. 1994; Wolfsperger et al. 1994). In the present study, we were unable to associate exposure with hair As levels, and it is difficult to discern how hair could become an alternative biometric for examining arsenic body burden at moderately elevated exposure conditions on a population or individual basis.

The Korean community consumes food-stuffs on a daily basis that contain As, such as rice, seaweed, and shellfish. Chicken may also be of concern in some populations, but the consumption rate in this community (21 g/day) is but a third of the average daily intake observed nationally in the United States (60 g/day) (Lasky et al. 2004). The consumption rates for foodstuffs (Table 2) led to an estimated total As intake approximately equal to the estimated urine total As excretion rate. Although urinary excretion of As ingested is not complete and may underestimate the total amount ingested because of a portion of the As body burden being contained in and excreted via hair and skin, the mass balance results suggest that these food items will represent the primary sources of exposure for total As and accordingly, for inorganic As. It should be noted that some of the data used to calculate mass balance were from studies with small sample size, unknown species type, and different methodologies (e.g., raw vs. cooked, dry weight vs. wet weight), and these variables could not always be controlled for in this study. Accordingly, the choice of studies selected for determining the median total As value and/or the possibility that we did not obtain all the literature values could facilitate an alteration in the total As intake.

In this population, the average fraction of the total As in urine comprising the sum of As species is 25%, or 48 μg/day (Table 4). Of this 48 μg/day, the shellfish As data obtained for the study indicate that only a small fraction (approximately 4%; 2 μg/day) comes from the consumption of shellfish (mollusks). Exposure to inorganic As, DMA, and MMA from finfish, crab, shrimp, and seaweed was not addressed in this study, as the available data are insufficiently robust to allow for an approximation. These sources, along with others discussed (drinking water, rice, clams, and to a lesser extent, chicken), should represent the vast majority of the As intake that is excreted in urine as the sum of As species. A rough calculation based on intake values used or derived in this study indicates that drinking water could account for 3 μg/day of inorganic As (2 μg/L × 1.5 L/day), and rice for approximately 16 μg/day of inorganic As (160 g/day × 0.1 μg/g). Chicken could possibly contribute 2 μg/day inorganic As (21 g/day × 0.1 μg/g), and shellfish (mollusks) about 2 μg/day as sum of As species. The sum of these intakes would represent approximately half of the total sum of As species intake of 48 μg/day, based on urinary As levels. The remaining half may come from crab, shrimp, finfish, seaweed, and possibly other unidentified sources, or the values used in this calculation may underestimate actual intake levels.

## Conclusion

The primary sources of exposure to As within this community may come from several food sources, with rice consumption possibly being an important source of inorganic As. Clam consumption is not. Rice has higher levels of arsenic (and inorganic As) compared with many crops and can contribute to the total daily intake of arsenic (and inorganic As) in populations consuming rice in quantity (Meharg et al. 2008; Meharg and Rahaman 2003; Williams et al. 2005, 2006, 2007; Zavala and Duxbury 2008; Zavala et al. 2008). Also, recent evidence suggests that rice may be divided into two distinct categories, with the As found in one type being readily less absorbed and, once absorbed, excreted more rapidly than in the other type (Zavala et al. 2008). As the consumption of rice may provide a significant source of exposure to inorganic As, we need to improve our understanding of the extent to which As is biologically available and once available, determine the toxicologic significance of the fraction that is bioavailable.

This Korean community may have average urinary sum of As species levels that place them within the highest 5th percentile of the NHANES distribution representing the U.S. population. Further, they may have dietary behaviors that result in a portion of the community having elevated inorganic As exposure. However, their exposure is less than that associated with populations exposed to high levels of inorganic As from drinking water (> 100 μg/L).

## Figures and Tables

**Table 1:** Total As, inorganic As (sum As^+5^ and As^+3^), and DMA levels for shellfish species based on individual and composite samples.

	Littleneck clam		Butter clam		Oyster		Varnish clam^a^ composite	Eastern softshell clam		Horse clam^b^		
	Individual	Composite	Individual	Composite	Individual	Composite		Siphon	Viscera	Siphon^c^	Viscera^d^	Composite
Total As [μg/g (SD)]	3.7 (2.3)	2.2 (0.8)	4.9 (1.4)	3.2 (1.2)	1.3 (0.4)	1.2 (0.4)	1.7 (0.2)	1.2 (0.8)	1.8 (0.8)	1.4 (0.5)	1.3 (0.1)	0.9 (0.12)
Inorganic As [μg/g (SD)]	0.08 (0.03)	0.04 (0.03)	0.06 (0.01)	0.04 (0.03)	0.02 (0.02)	0.01 (0.01)	0.02 (0.01)	0.09 (0.06)	0.06 (0.03)	0.12 (0.08)	0.06 (0.08)	0.04 (0.02)
DMA [μg/g (SD)]	0.15 (0.11)	0.11 (0.05)	0.17 (0.09)	0.14 (0.05)	0.08 (0.03)	0.08 (0.02)	0.05 (0.02)	0.04 (0.02)	0.11 (0.06)	0.05 (0.02)	0.11 (0.05)	0.06 (0.02)
No.	11	16	11	17	11	11	4	8	8	16–18	15–17	3
No. in each composite	NA	10–13	NA	10–13	NA	11	11	NA	NA	NA	NA	13

NA, not available. Values shown are the sample size for each species (SD around the means) along with the number or range of individuals in each composite.

a Individual values were not determined for varnish clams; these clams were found only at one location.

b Composite values are for whole immature animals; individual siphon and viscera values are from large mature animals.

c We used 18 animals to obtain siphon data; 2 horse clam samples were destroyed in processing for deriving total As (*n* = 16), while all animals were used to derive inorganic As value.

d We used 18 animals to obtain viscera data; 2 horse clam samples were destroyed in processing for deriving total As, and one horse clam was not completely obtained in the field (*n* = 15). All animals were used to obtain inorganic value with the exception of the clam not completely obtained in the field (*n* = 17).

**Table 2:** Estimated mean daily total As intake.^a^

Food item	Total As (μg/g) range from literature^b^	Total As point estimate (μg/g)	Mean consumption rate [g/day ± SD]	Estimated total As intake (μg/day)
Crab	1.5–17.4	3.7	5.7 ± 10.1	21
Shrimp	1.1–20.8	1.9	3.7 ± 9.2	7
Shellfish (nine clam species)	NA	NA	15.1 ± 21.8	39^c^
Sum of crab, shrimp, and nine clam species	NA	NA	24.4 ± 30.2	67
	NA	NA	24.8 ± 30.4^d^	NA
Sum of all shellfish species				
Rice	0.1–0.6	0.24	163.4 ± 157.5	39
Seaweed	0.2–18.1	3.4^e^	3.1 ± 4.4	10
Finfish	NA	NA	62.9 ± 63.0	79
			Sum total As intake^f^	195
			Mean urinary As (μg/day)^g^	191

NA, not available.

a Estimated mean daily total As intake was based on consumption of crab, shrimp, shellfish (mollusks), rice, seaweed, and finfish including SDs for 89 participants from within the Korean cohort (*n* = 108) along with mean total daily As excreted in urine for the 67 participants who provided samples; *n* = 89 is based on total daily As for each individual for whom all data were available (one individual did not complete the FFQ) and who reported consuming > 600 Kcal/day (*n* = 18 reported consuming < 600 Kcal/day). Total As represents sum of As species (DMA, MMA, As^+3^ and As^+5^ combined) and AsB plus other unidentified forms of As species.

b Citations are provided in “Methods.”

c Total As levels from tissue sample data (see Table 1) were used along with values for three other clam species consumed (geoduck, mussels, and razor clam). Additional details are provided in “Methods.”

d This mean consumption rate of 25 g/day for *n* = 89 is slightly but not significantly higher than the 23 g/day value for the total sample (*n* = 108) (Tsuchiya et al. 2008a).

e Total As levels for the seaweed species *Hizikie fusiforme* were not included. Although this species is known to have high levels of total As, this species is not consumed by this population (see “Methods”).

f Based on individual values from the category “Sum of crab, shrimp and nine clam species” and not “Sum of all shellfish species.”

g Mean total daily As excreted (μg/day) is based on the unadjusted mean from Table 2 (127.3 μg/L) and the excretion rate of 1.5 L/day.

**Table 3:** Creatinine-normalized and unadjusted As levels in urine for the 67 participants who provided samples from the Korean cohort (*n* = 108).

	Adjusted (μg/g creatinine)					Unadjusted (μg/L)				
	Sum As species^a^	Total As^b^	As^+3^ and As^+5^	DMA	MMA	Sum As species	Total As	As^+3^ and As^+5^	DMA	MMA
GM^c^	27.1	104.3	NA	NA	NA	21.8	78.1	NA	NA	NA
Mean	37.2	152.1	2.9	33.0	1.5	30.6	127.3	2.4	27.0	1.2
SD	25.0	162.1	3.5	24.2	2.3	26.7	156.6	3.9	25.3	2.0
5th	7.4	21.8	< LOQ	6.5	< LOQ	7.1	17.5	< LOQ	5.5	< LOQ
50th	36.3	98.6	0.9	28.1	< LOQ	21.6	75.0	0.8	19.8	< LOQ
95th	75.8	387.5	9.7	62.2	6.5	90.4	326.6	10.8	77.0	6.0

Abbreviations: < LOQ, less than limit of quantification; NA, not available. 5th, 50th, and 95th are percentiles.

a Sum of As species is DMA, MMA, and As^+3^ and As^+5^ combined.

b Total As was determined also and represents sum of As species and AsB plus other unidentified forms of As species.

c GM values were not determined, as 33, 1, and 40 participants had analytical MMA, DMA, and As^+3^ and As^+5^ levels, respectively, below the limit of detection.

**Table 4:** Estimated mean total daily intake of shellfish, estimated mean intake of As species, and mean sum of As species in urine.^a^

	Korean cohort	Korean cohort providing urine sample
Mean intake of nine species of clams consumed [g/day (SD)]^b^	13.5 (20.2)	12.9 (19.5)
Mean As species intake from nine species of clams [μg/day (SD)]^c^	2.0 (2.7)	1.7 (2.5)
Sum of species in urine [μg/day (SD)]^d^	NA	47.9 (40.9)

NA, not available.

a Estimated mean total daily intake of shellfish (mollusks) species consumed and estimated mean intake of As species are provided for the Korean cohort (*n* = 108) and for those who provided urine samples (*n* = 67); the mean sum of As species in micrograms excreted daily in urine is provided only for the 67 participants who provided samples.

b Shellfish species consumption in grams per day (± SD) based on intake of six shellfish species collected along with geoduck, mussels, and razor clam.

c As species include DMA, MMA, and As^+3^ and As^+5^ combined.

d Sum of As species includes DMA, MMA, and As^+3^ and As^+5^ and is based on the unadjusted value from Table 2 (30.6 μg/L) and an excretion rate of 1.5 L/day.

## References

[b1-ehp-117-632] ACGIH (American Conference of Governmental Industrial Hygienists) (2001). Variability in measurements in urine. Documentation of the Biological Exposure Indices.

[b2-ehp-117-632] Andrén P, Schütz A, Vahter M, Attewell L, Johansson L, Willers S (1988). Environmental exposure to lead and arsenic among children living near a glassworks. Sci Total Environ.

[b3-ehp-117-632] ATSDR (Agency for Toxic Substances and Disease Registry) (U.S.) (2007). Toxicological profile for arsenic.

[b4-ehp-117-632] Binder S, Forney D, Kaye W, Paschal D (1987). Arsenic exposure in children living near a former copper smelter. Bull Environ Contam Toxicol.

[b5-ehp-117-632] Buchet JP, Staessen J, Roels H, Lauwerys R, Fagard R (1996). Geographical and temporal differences in the urinary excretion of inorganic arsenic: a Belgian population study. Occup Environ Med.

[b6-ehp-117-632] Caldwell KL, Jones RL, Verdon JC, Jarrett JM, Caudill SP, Osterloh JD (2008). Levels of urinary total and speciated arsenic in the US population: National Health and Nutrition Examination Survey 2003–2004. J Exp Sci Environ Epidemiol.

[b7-ehp-117-632] Chew CM (1996). Toxicity and Exposure Concerns Related to Arsenic in Seafood: An Arsenic Literature Review for Risk Assessments.

[b8-ehp-117-632] Chiou HY, Hsueh YM, Hsieh LL, Hsu LI, Hsu YH, Hsieh FI (1997). Arsenic methylation capacity, body retention, and null genotypes of glutathione *S*-transferase M1 and T1 among current arsenic-exposed residents in Taiwan. Mutat Res.

[b9-ehp-117-632] Das D, Chatterjee A, Mandal BK, Samanta G, Chakraborti D, Chanda B (1995). Arsenic in ground water in six districts of West Bengal, India: the biggest arsenic calamity in the world. Part 2. Arsenic concentration in drinking water, hair, nails, urine, skin-scale and liver tissue (biopsy) of the affected people. Analyst.

[b10-ehp-117-632] Foà V, Colombi A, Maroni M, Buratti M, Calzaferri G (1984). The speciation of the chemical forms of arsenic in the biological monitoring of exposure to inorganic arsenic. Sci Total Environ.

[b11-ehp-117-632] Gottlieb K, Koehler JR, Tessari J (1993). Non-analytic problems in detecting arsenic and cadmium in children living near a cadmium refinery in Denver, Colorado. J Exp Anal Environ Epidemiol.

[b12-ehp-117-632] Harkins DK, Susten AS (2003). Hair analysis: exploring the state of the science. Environ Health Perspect.

[b13-ehp-117-632] Hsiung T-M, Huang C-W (2006). Quantitation of toxic arsenic species and arsenobetaine in pacific oysters using an offline process with hydride generation-atomic absorption spectroscopy. J Agric Food Chem.

[b14-ehp-117-632] Hwang Y-H, Bornschein RL, Grote J, Menrath W, Roda S (1997). Urinary arsenic excretion as a biomarker of arsenic exposure in children. Arch Environ Health.

[b15-ehp-117-632] Jensen GE, Christensen JM, Poulsen OM (1991). Occupational and environmental exposure to arsenic. Increased urinary arsenic level in children. Sci Total Environ.

[b16-ehp-117-632] Kalman DA (1988). Quantitation of arsenic species in urine for exposure assessment studies. J Res Natl Bur Stand.

[b17-ehp-117-632] Kalman DA, Hughes J, van Belle G, Burbacher T, Bolgiano D, Coble K (1990). The effect of variable environmental arsenic contamination on urinary concentrations of arsenic species. Environ Health Perspect.

[b18-ehp-117-632] Kapaj S, Peterson H, Liber K, Bhattacharya P (2006). Human health effects from chronic arsenic poisoning—a review. J Environ Sci Health A Tox Hazard Subst Environ Eng.

[b19-ehp-117-632] Kavanagh P, Farago ME, Thornton I, Goessler W, Kuehnelt D, Schlagenhaufen C (1998). Urinary arsenic species in Devon and Cornwall residents, UK. A pilot study. Analyst.

[b20-ehp-117-632] Kolar AS, Patterson RE, White E, Neuhouser ML, Frank LL, Standley J (2005). A practical method for collecting 3-day food records in a large cohort. Epidemiology.

[b21-ehp-117-632] Koons RD, Peters CA (1994). Axial distribution of arsenic in individual human hairs by solid sampling graphite furnace AAS. J Anal Toxicol.

[b22-ehp-117-632] Kristal AR, Beresford SA, Lazovich D (1994). Assessing change in diet-intervention research. Am J Clin Nutr.

[b23-ehp-117-632] Kristiansen J, Christensen JM, Iversen BS, Sabbioni E (1997). Toxic trace element reference levels in blood and urine: influence of gender and life-style factors. Sci Total Environ.

[b24-ehp-117-632] Kurttio P, Komulainen H, Hakala E, Kahelin H, Pekkanen J (1998). Urinary excretion of arsenic species after exposure to arsenic present in drinking water. Arch Environ Contam Toxicol.

[b25-ehp-117-632] Landolt ML, Hafer FR, Nevissi A, Van Belle G, Van Ness K, Rockwell C (1985). Potential Toxicant Exposure among Consumers of Recreationally Caught Fish from Urban Embayments of Puget Sound. NOAA Technical Memorandum NOS OMA 23.

[b26-ehp-117-632] Lasky T, Sun W, Kadry A, Hoffman MK (2004). Mean total arsenic concentrations in chicken 1989–2000 and estimated exposures for consumers of chicken. Environ Health Perspect.

[b27-ehp-117-632] MacIntosh DL, Williams PL, Hunter DJ, Sampson LA, Morris SC, Willett WC (1997). Evaluation of a food frequency questionnaire-food composition approach for estimating dietary intake of inorganic arsenic and methylmercury. Cancer Epidemiol Biomarkers Prev.

[b28-ehp-117-632] Mariën K, Patrick GM (2001). Exposure analysis of five fish-consuming populations for overexposure to methylmercury. J Expo Anal Environ Epidemiol.

[b29-ehp-117-632] Meharg AA, Deacon C, Campbell RCJ, Carey A-M, Williams PN, Feldmann J (2008). Inorganic arsenic levels in rice milk exceed EU and US drinking water standards. J Environ Monit.

[b30-ehp-117-632] Meharg AA, Rahaman M (2003). Speciation and localization of arsenic in white and brown rice grains. Environ Sci Technol.

[b31-ehp-117-632] Mohri T, Hisanaga A, Ishinishi N (1990). Arsenic intake and excretion by Japanese adults: a seven day duplicate diet study. Food Chem Toxicol.

[b32-ehp-117-632] Morse DL, Harrington JM, Housworth J, Landrigan PJ, Kelter A (1979). Arsenic exposure in multiple environmental media in children near a smelter. Clin Toxicol.

[b33-ehp-117-632] Narang APS, Chawla LS, Khurana SB (1987). Levels of arsenic in Indian opium eaters. Drug Alcohol Depend.

[b34-ehp-117-632] NRC (National Research Council) (1999). Arsenic in Drinking Water.

[b35-ehp-117-632] OEHHA (Office of Environmental Health Hazard Assessment) (2001). Chemicals in Fish: Consumption of fish and Shellfish in California and the United States Final report.

[b36-ehp-117-632] Olguín A, Jauge P, Cebrián M, Albores A (1983). Arsenic levels in blood, urine, hair, and nails from a chronically exposed human population. Proc West Pharmacol Soc.

[b37-ehp-117-632] Ostrom T, Williams P, Palcisko G (2007). Assessment of trace metals in tissues of geoduck clams from eastern Puget Sound. http://www.engr.washington.edu/epp/psgb/2007psgb/2007proceedings/papers/2e_ostr.pdf.

[b38-ehp-117-632] Patterson RE, Kristal AR, Tinker LF, Carter RA, Bolton MP, Agurs-Collins T (1999). Measurement characteristics of the Women’s Health Initiative food frequency questionnaire. Ann Epidemiol.

[b39-ehp-117-632] Paulsen G, Mai S, Zellmer U, Alsen-Hinrichs C (1996). Blood and hair arsenic, lead and cadmium analysis of adults and correlation analysis with special reference to eating habits and other behavioral influences [in German]. Gesundheitswesen.

[b40-ehp-117-632] Pollisar L, Lowry-Cobb K, Kalman DA, Hughes JP, van Belle G, Cover DS (1990). Pathways of human exposure to arsenic in a community surrounding a copper smelter. Environ Res.

[b41-ehp-117-632] Raie RM (1996). Regional variation in As, Cu, Hg, and Se and interaction between them. Ecotoxicol Environ Saf.

[b42-ehp-117-632] Sakai T, Wilbur S (2006). Routine Analysis of Toxic Arsenic Species in Urine using HPLC with ICP-MS. http://www.chem.agilent.com/Library/applications/5989-5505EN.pdf.

[b43-ehp-117-632] Smith TJ, Crecelius EA, Reading JC (1977). Airborne arsenic exposure and excretion of methylated arsenic compounds. Environ Health Perspect.

[b44-ehp-117-632] Takagi YS, Matsuda S, Imai S, Ohmori Y, Masuda T, Vinson JA (1988). Survey of trace elements in human nails: an international comparison. Bull Environ Contam Toxicol.

[b45-ehp-117-632] Toy KA, Polissar NL, Liao S, Mittelstaedt GD (1996). A Fish Consumption Study of the Tulalip and Squaxin Island Tribes of the Puget Sound Region.

[b46-ehp-117-632] Trepka MJ, Heinrich J, Schulz C, Krause C, Popescu M, Wjst M (1996). Arsenic burden among children in industrial areas of eastern Germany. Sci Total Environ.

[b47-ehp-117-632] Tsuchiya A, Hardy J, Burbacher TM, Faustman EM, Mariën K (2008a). Fish intake guidelines: incorporating n-3 fatty acid intake and contaminant exposure in the Korean and Japanese communities. Am J Clin Nutr.

[b48-ehp-117-632] Tsuchiya A, Hinners T, Burbacher TM, Faustman EM, Mariën K (2008b). Mercury exposure from fish consumption within the Japanese and Korean communities. J Toxicol Environ Health.

[b49-ehp-117-632] U.S. EPA (U.S. Environmental Protection Agency) (1992). Method 200.3 sample preparation procedure for spectrochemical determination of total recoverable elements in biological tissues. Methods for the Determination of Metals in Environmental Samples.

[b50-ehp-117-632] U.S EPA (U.S. Environmental Protection Agency) (2006). Drinking Water Contaminants.

[b51-ehp-117-632] U.S EPA (U.S. Environmental Protection Agency) (2007). Integrated Risk Information System.

[b52-ehp-117-632] Vahter M, Concha G, Nermell B, Nilsson R, Dulout F, Natarajan AT (1995). A unique metabolism of inorganic arsenic in native Andean women. Eur J Pharmacol.

[b53-ehp-117-632] Vahter M, Lind B (1986). Concentrations of arsenic in urine of the general population in Sweden. Sci Total Environ.

[b54-ehp-117-632] Valentine JL, Kang HK, Spivey G (1979). Arsenic levels in human blood, urine, and hair in response to exposure via drinking water. Environ Res.

[b55-ehp-117-632] Valkonen S, Aitio A, Jarvisalo J, Brätter P, Schramel P (1983). Urinary arsenic in a Finnish population without occupational exposure to arsenic. Trace Elelment Analytical Chemistry in Medicine and Biology.

[b56-ehp-117-632] Vienna A, Capucci E, Wolfsperger M, Hauser G (1995). Heavy metals in hair of students in Rome. Anthropol Anz.

[b57-ehp-117-632] Wang CT, Chang WT, Huang CW, Chou SS, Lin CT, Liau SJ (1994). Studies on the concentrations of arsenic, selenium, copper, zinc, and iron in the hair of blackfoot disease patients in different clinical stages. Eur J Clin Chem Clin Biochem.

[b58-ehp-117-632] Willett W (1998). Nutritional Epidemiology.

[b59-ehp-117-632] Willams PN, Islam MR, Adomako EE, Raab A, Hossain SA, Zhu YG (2006). Increase in rice grain arsenic for regions of Bangladesh irrigating paddies with elevated arsenic in groundwaters. Environ Sci Technol.

[b60-ehp-117-632] Williams PN, Price AH, Raab A, Hossain SA, Feldmann J, Meharg AA (2005). Variation in arsenic speciation and concentration in paddy rice related to dietary exposure. Environ Sci Technol.

[b61-ehp-117-632] Williams PN, Villada A, Deacon C, Raab A, Figuerola J, Green AJ (2007). Greatly enhanced arsenic shoot assimilation in rice leads to elevated grain levels compared to wheat and barley. Environ Sci Technol.

[b62-ehp-117-632] Wolfsperger M, Hauser G, Gössler W, Schlagenhaufen C (1994). Heavy metals in human hair samples from Austria and Italy: influence of sex and smoking habits. Sci Total Environ.

[b63-ehp-117-632] Yamauchi H, Takahashi K, Mashiko M, Yamamura Y (1989). Biological monitoring of arsenic exposure of gallium arsenide- and inorganic arsenic-exposed workers by determination of inorganic arsenide and its metabolites in urine and hair. Am Ind Hyg Assoc J.

[b64-ehp-117-632] Zavala YJ, Duxbury JM (2008). Arsenic in rice: I. Estimating normal levels of total arsenic in rice grain. Environ Sci Technol.

[b65-ehp-117-632] Zavala YJ, Gerads R, Gürleyük H, Duxbury JM (2008). Arsenic in rice: II. Arsenic speciation in USA grain and implications for human health. Environ Sci Technol.

